# Ethnic Diversity and Students’ Social Adjustment in Dutch Classrooms

**DOI:** 10.1007/s10964-021-01507-y

**Published:** 2021-10-08

**Authors:** Anke Munniksma, Johanna Ziemes, Philipp Jugert

**Affiliations:** 1 Department of Child Development and Education, University of Amsterdam, Amsterdam, The Netherlands; 2 Department of Educational Science, University of Duisburg-Essen, Essen, Germany; 3 Department of Psychology, University of Duisburg-Essen, Essen, Germany

**Keywords:** Classroom diversity, Social adjustment, Group differences, Secondary schools, Adolescents, Open classroom climate for discussion

## Abstract

Research in the US indicates that classroom diversity is related to better social adjustment of students, but research on this association in European classrooms is limited in scope and yields inconsistent findings. This study examined how classroom ethnic diversity is related to social adjustment of societally dominant versus minoritized ethnic groups, and how an open classroom climate for discussion contributes to this. This was examined in low to moderately diverse Dutch classrooms (2703 secondary school students, from 119 classrooms and schools, M_age_ = 14, 50% female, 18% foreign-born parents). Results revealed that students from minoritized groups reported lower social adjustment. For all students, classroom ethnic diversity was related to worse social adjustment which was partly explained by classroom socioeconomic status (SES). An open classroom climate for discussion did not moderate the relation between diversity and social adjustment. The findings indicate that students’ social adjustment is worse in ethnically diverse and low-SES classrooms, and an open classroom climate for discussion does not solve this.

## Introduction

The last decades have been characterized by an increase in ethnic diversity in Western societies, and it is expected to continue to increase (Eurostat, 2021; Jennissen et al., 2018, U.S. Census, 2020). This ethnic diversity is also present among the school-aged population, meaning that more students experience ethnic diversity within their classrooms nowadays. Diversity means that there are more different (ethnic) groups represented in a context (Simpson, 1949), and this greater diversity allows for greater numerical balance among different ethnic groups (Graham, 2018). The question is how classroom ethnic diversity affects the lives of youth. In this regard, previous research in both the US and Europe has examined associations between school ethnic diversity and a variety of outcomes like academic achievement (e.g., Van Ewijk & Sleegers, 2010) mental health (see for a review: DuPont-Reyes & Villatoro, 2019) and intergroup relations (see for an overview: Thijs & Verkuyten, 2014). Whereas several studies in the US showed that classroom diversity was also related to better social adjustment at school (e.g., Graham, 2018), research on this association in Europe is scarce, focused on only one aspect of social adjustment (i.e., bullying), and results are mixed (Vitoroulis et al., 2016; Özdemir et al.,^2018^). In combination with different operationalizations of ethnic diversity and unrepresentative sampling, this limits generalizability of these findings in Europe.

The current study moves beyond examining the association of classroom ethnic diversity with social adjustment, to the question under which conditions diversity is beneficial and for whom (cf. Brown & Juvonen, 2018). Regarding “for whom”, this study examines whether the relation between classroom diversity and students’ social adjustment differs between ethnic groups in society. As ethnic groups, the societally dominant ethnic group (i.e., students without a migration history) versus societally minoritized ethnic groups (students with at least one parent born abroad) are distinguished. Next, regarding “under which conditions”, this study looks into the moderating role of an open classroom climate for discussion, by drawing from the citizenship education literature. As such, the current study aims to answer the following research questions: (1) Is classroom ethnic diversity related to students’ social adjustment differently for members of societally dominant vs. minoritized groups? And, (2) is the relation between classroom ethnic diversity and students’ social adjustment moderated by an open classroom climate for discussion? This will be examined using a nationally representative sample, of over 2700 second-year secondary school students in 119 classrooms in an equal number of schools (i.e., one classroom per school) in the Netherlands. Reflecting the ethnic compositions of Dutch classrooms, this sample includes mainly low to moderately diverse classrooms.

### Students’ Social Adjustment at School

Social adjustment refers to students’ social and affective experiences, which in the school context involves to what extent students feel well and safe in the school environment, and get along with their peers as opposed to being victimized by them (e.g., being bullied). These social experiences are often more turbulent during adolescence, as peers become more influential at this age (Brechwald & Prinstein, 2011), while peer victimization peaks during these years (Rivara & Le Menestrel, 2016). Also, research shows that members of societally minoritized ethnic groups - students with a migration history - often report worse social adjustment at school than members of the societally dominant ethnic group (Berkowitz, 2020; Stevens et al., 2020) and that boys are more commonly victimized than girls (Card et al., 2007). However, previous research shows that positive social adjustment at school is important because negative social adjustment is related to lower academic engagement and achievement (Olivier et al., 2018). Hence, in order to promote social equality, positive social adjustment for all students is important.

### Classroom Ethnic Diversity and Social Adjustment of Minoritized Group Students

Next to differences between societally dominant and minoritized ethnic group members, school and classroom level diversity may affect the nature of social relations within classrooms, and as such students’ social adjustment. Several theories shed light on the relation between ethnic diversity and social adjustment leading to different hypotheses for members of societally dominant ethnic groups and members of societally minoritized groups. Focusing on societally minoritized group members, the *balance of power hypothesis* (Juvonen et al., 2006) has been introduced to explain how diversity may affect students’ social adjustment within classrooms. According to the proposed balance of power mechanism, the social dynamics in a group will be more positive when groups are more balanced in size. This is the case in classrooms that are marked by greater ethnic diversity, as ethnic diversity refers to the number of different groups and to what extent these groups are balanced in size (Simpson, 1949). Juvonen and colleagues (2006) argued that when different ethnic groups are similar in size, they are similar in (social) power. Combined with the widely used definition of bullying (Olweus, 1994) which states that there is an imbalance of power between the perpetrator and the victim, this implies that when there is no power imbalance, there would be less bullying, and as such better social adjustment. On the other hand, when there is an imbalance of power (i.e., unequal group sizes), then this is often to the disadvantage of societal minoritized group members who are often also the numerical minority students within classrooms. As such, in classrooms with an unequal distribution of ethnic groups, societally minoritized group students would more often be the victims of bullying due to their lower power position, and as such have less positive social experiences at school. Hence, particularly members of societally minoritized groups would benefit from classroom diversity in terms of their social adjustment.

In line with this reasoning, research on social adjustment from the US (metropolitan Los Angeles) showed that higher classroom diversity was related to greater sense of safety, less harassment, less feelings of loneliness among sixth-grade (aged 11) African-American and Latino students (Juvonen et al., 2006, 2018). It should be noted that this research made use of purposeful sampling to ensure representation of classrooms with different levels of ethnic diversity. Also among older students in California (Grade 9 and 11) school diversity was related to less peer victimization among high school (Felix & You, 2011). Another study among high school students (Grade 9) in Virginia found that classroom diversity and percentage of minoritized students were not related to students’ reported bullying, but diversity was related to less perceived bullying by teachers and percentage of minoritized students was related to more teacher perceived bullying (Klein & Cornell, 2010).

In Europe, several studies documented that a higher share of minoritized group students was related to better social adjustment, particularly less victimization, of students from minoritized ethnic groups. For example, students from minoritized groups (aged 12) in Flanders reported less peer victimization in primary schools with a higher proportion of minoritized students (Agirdag et al., 2011). Similarly, first generation immigrant fifteen-year-old students in Sweden were less often exposed to bullying in secondary schools with higher (rather than lower) shares of minoritized students (Hjern et al., 2013). Concerning ethnic bullying, a higher percentage of primary school students from minoritized groups was related to fewer experiences of peer victimization among minoritized students (aged 10–12) in the Netherlands (Verkuyten & Thijs, 2002). Additionally, one study including 11 countries (incl. European countries and the US) found that in all countries the share of minoritized students in school was related to less peer victimization among minoritized students (aged 11–15; Walsh et al., 2016). Focusing on more aspects of social adjustment, one study documented that minoritized youth (aged 14–15) in Swedish secondary school classrooms with a high share of minoritized students were less often rejected, isolated, and victimized than in classrooms with low shares of minoritized students (Plenty & Jonsson, 2017). Looking into the association of specifically ethnic diversity (i.e., more groups and more balanced group sizes) and friendships of students, one large scale study at secondary schools (third and fifth grade) in Flanders documented that higher school ethnic diversity was related to higher quantity and quality of friendships (regardless of school SES) of minoritized group students (Demanet et al., 2012) but a study among secondary schools in one Dutch city (students age 12) found that classroom diversity was not related to the quantity of friendships with classmates for students from minoritized ethnic groups (Munniksma et al., 2017a).

Taken together, whereas the European studies indicate that the percentage of societally minoritized group students reduces bullying and victimization among societally minoritized group students, the studies on (specifically) ethnic diversity and friendships show contrasting results. Nonetheless, the findings regarding the share of students from minoritized groups do shed some light on the potential benefits of classroom diversity, because in these countries a higher percentage of minoritized group students often means that ethnic groups are more balanced in size, which is one of the indicators of diversity (see high correlation, *r* = 0.92, between ethnic diversity and percentage of societally minoritized group members in Dutch classrooms in e.g. Munniksma et al., 2017a). Hence, if this is also the case in the representative sample of the current study, one would expect that the findings regarding the correlates of percentage minoritized group students and classroom diversity would be comparable.

### Classroom Ethnic Diversity and Social Adjustment of Societally Dominant Group Students

Focusing on students from the societally dominant ethnic group, *ethnic competition theory* (Blalock, 1967; Scheepers et al., 2002) assumes that when people in a given context are confronted with more ethnic diversity this will elicit feelings of threat. Ethnic diversity may be threatening to societally dominant group members because it goes along with a decreased share of same-ethnic peers and may jeopardize the high-status position of the societally dominant ethnic group in contexts where they are in a numerical minority position. While members of societally minoritized groups are used to being in a position of lower power (i.e., lower ingroup representation within a given context and lower societal status), this situation may be new and uncomfortable for members of the societally dominant ethnic group. Societally dominant group members may react to this by excluding the other group(s), and turning more to their ingroup (see Munniksma et al., 2017a). When students do not form social relationships with part of the classroom (i.e., outgroup members), this (i.e., segregated social networks) is likely to negatively affect students’ social adjustment in the classroom. Based on ethnic competition theory (Blalock, 1967; Scheepers et al., 2002) one would thus expect that classroom ethnic diversity would be negatively related to the social adjustment of societally dominant group members.

Research in the US, on the relation between classroom diversity and social adjustment of societally dominant group students, does not support this reasoning. School and classroom diversity was related to more positive social adjustment for minoritized as well as dominant group members in the US (aged 11; Juvonen et al., 2018). In contrast, there is more support for ethnic competition theory in European studies that primarily focus on bullying. In these studies, a higher share of students from minoritized groups at school was related to more bullying among classmates. This was shown in a series of studies conducted in the Netherlands, among both minoritized and dominant ethnic group preschoolers (ages 5–6) in elementary schools (Jansen et al., 2016), among minoritized and dominant group students (aged 11) in the last year of elementary school (Tolsma et al., 2013), and in a study among 13-year-old middle school students (aged 12; Vervoort et al., 2010). In UK elementary schools, a higher proportion of societal minority group students was related to more discriminatory aggression for both minoritized and dominant group students (aged 8–12; Durkin et al., 2012). Also in 11 countries, a higher share of immigrant students at school was related to higher levels of physical fighting and bullying perpetration for both minoritized and dominant group students (aged 11–15; Walsh et al., 2016). Another study among secondary school students (aged 14) in Sweden documented that classroom ethnic diversity was related to majority students engaging in more ethnic harassment (Özdemir et al., 2018). Together, these studies support ethnic competition theory.

Regarding positive aspects of social adjustment, (specifically) ethnic diversity was related to lower quantity and quality of friendships of majority group secondary school students in Flanders (Demanet et al., 2012), but this could be attributed to the schools’ socio-economic situation (measured by mean parental occupational status per school). In contrast, ethnic diversity did not affect the total number of friendships of majority (and minority) students (aged 12) in classrooms in secondary schools in one Dutch city (Munniksma et al., 2017a). In sum, previous findings are inconclusive, the primary focus is on negative aspects of social adjustment, and few studies examined ethnic diversity specifically.

As illustrated by the study of Demanet and colleagues (2012), it is important to consider classroom socio-economic status (SES) when examining correlates of classroom ethnic diversity. Ethnically diverse schools may be the schools that are socially disadvantaged (i.e., students from families with lower SES), which may also yield lower social adjustment of students within these schools due to SES differences in social adjustment (see e.g., Marçal, 2020). Such social differences between schools and classrooms may be more pronounced in countries with tracked educational systems like the Netherlands, where students are selected into different educational tracks at the start of secondary school (at age 12). In the Netherlands, students from minoritized ethnic groups -with a migration background- and students with a lower SES are overrepresented in lower educational tracks (Vogels et al., 2021). Hence, the study of outcomes of classroom diversity should include the classrooms’ socio-economic situation (average SES) to asses that diversity effects are not in fact attributable to classroom SES effects.

### How Can Positive Outcomes of Classroom Diversity be Encouraged?

Several studies show that outcomes of diversity can be promoted by learning about the outgroup and by taking the perspective of outgroups (Pettigrew & Tropp, 2008). One way to encourage this can be found in the citizenship education literature. In this field of study, an *open classroom climate for discussion* is considered important in promoting civic engagement (Deimel et al., 2020), and positive intergroup attitudes (Janmaat, 2012). In such an open classroom climate for discussion (Torney-Purta et al., 2001) the teacher provides space for discussion, encourages different perspectives, and students learn from different perspectives. This suggests that schools should be seen as small societies and that students learn the rules of social interaction not just through explicit teaching (i.e., civics classes) but also through informal learning (i.e., classroom climate for discussion). Further, previous research shows a positive relationship between open classroom climate for discussion and tolerance towards immigrants (Diedrich, 2006; Gniewosz & Noack, 2008), suggesting that for diversity to exert positive effects for all, it needs to be coupled with an open classroom climate for discussion. Because an open classroom climate for discussion gives insight into different perspectives and thereby allows students to learn about other ethnic groups that are present within a classroom, it can be expected that that classroom ethnic diversity is more positively related to social-emotional experiences in classrooms with an open classroom climate for discussion.

## Current Study

The main goal of this study was to investigate whether and how ethnic diversity at the classroom level is related to social adjustment of members of the dominant ethnic group versus members of minoritized ethnic groups in society. Based on the balance of power mechanism, it was hypothesized that classroom diversity would be positively related to social adjustment for societally minoritized ethnic group students (*Hypothesis 1a*). In contrast, based on ethnic competition theory, it was hypothesized that classroom ethnic diversity would be negatively related to social adjustment for societally dominant ethnic group students (*Hypothesis 1b*). Drawing on insights from the citizenship education literature, it was hypothesized that classroom ethnic diversity would be more positively related to social adjustment in classrooms with a more open classroom climate for discussion (*Hypothesis 2*). All hypotheses of this study were pre-registered prior to analyses and are available online (https://osf.io/jbvus/?view_only=aa6d8582ea834593a305331de08eda5b). Hypotheses were tested in a nationally representative sample of 14-year old students in the Netherlands. This study examined two indicators of students’ social adjustment, representing negative and positive aspects: self-reported peer victimization and positive peer relations.

## Method

### Sample

The International Civic and Citizenship Education Study (ICCS) 2016 (see Schulz et al., 2018a) of the Netherlands is a suitable dataset to examine whether classroom diversity is related to social adjustment of youth at school. Countries had the liberty to design their assessment of ethnic diversity. The Dutch data (see Munniksma et al., 2017b) includes specific information on ethnic backgrounds of the students, allowing a detailed measure of diversity. In the Netherlands, 2812 second-year students (*m*_age_ = 14), in 123 classrooms, from an equal number of secondary schools participated in ICCS 2016. The overall sample consisted of 64 pre-vocational track, 55 general track, and 4 mixed-track classrooms (both pre-vocational and general). Due to power considerations, the four mixed-track classrooms were excluded from the analyses in this study. This resulted in an analytic sample of 2703 students, from 119 classrooms. Of the analytic sample, 50 percent of the students were female, and 18 percent of the students indicated that at least one of the parents was born abroad.

### Procedure

The ICCS data collection was conducted following strict guidelines (see Schulz et al., 2018b) to warrant nationally representative data and internationally comparable data. Sample selection followed a two-stage approach. In the first stage schools were selected within the countries based on probability proportional to size (PPS) and stratified by school type. In the second stage, one classroom (with all students), 15 teachers, and one school leader was selected at random per school. Data collection took place from February to April in 2016. Students completed a cognitive test (to asses civic knowledge), an international student questionnaire (to asses citizenship competences and aspects of citizenship education), and a European student questionnaire (with Europe specific measures). For the current study, data from the international student questionnaire were used.

### Main Measures

#### Experiences with Victimization

Students indicated their experiences with victimization by indicating how often six examples of abuse had happened to them at school in the past three months. Example items were: “A student called you by an offensive nickname”, “You were physically attacked by another student”, and “A student broke something belonging to you on purpose”. Students indicated how often this occurred on a scale from 1, *never*, to 4, *five or more times*. These six items together formed a reliable scale (Cronbach’s α = 0.71). A higher score on the scale indicates more experiences with victimization. As the mean scale of victimization was highly skewed (1.792), we dichotomized the items – zero indicating that students experienced no victimization and one that they experienced some. On this basis we created a sum scale which indicated how many aspects of victimization students experienced, and a binary variable which indicates if students experienced any form of victimization or not. The sum scale had a range from zero to five, a mean of 1.26 and a standard deviation of 1.30. The intra-class correlation (ICC) of the sum scale is 0.059. The ICC indicates the proportion of variance on the classroom level in contrast to the individual student level and has a theoretical range of 0 to 1 (see Lorah, 2018). Higher values indicate a larger amount of variance on the cluster level.

#### Positive Peer Relations

Students reported to what extent they agreed with statements about students in their school. The scale included four items about the nature of peer relationships at school: “Most students at my school treat each other with respect”, “Most students at my school get along well with each other”, “My school is a place where students feel safe”, and “I am afraid of being bullied by other students” (inverted). Students answered on a scale from 1, *strongly agree*, to 5, *strongly disagree*. The four items formed a scale (Cronbach’s α = 0.66). The scale was recoded so that a higher score means more positive peer relations. The ICC of the scale is 0.094.

#### Open Classroom Climate for Discussion

Students reported their perception of how open the classroom climate for discussion was by indicating for six statements how often this occurs when political or societal issues are discussed in class. Example statements are “Teachers encourage students to express their opinions”, and “Students express opinions in class even when their opinions are different from most of the other students”. Students answered from 1, *never*, to 4, *often*. A higher score indicates a more open perceived classroom climate for discussion. The six items form a reliable scale (Cronbach’s α = 0.76). The analyses use the scale provided within the international data file, which are based on Rasch-analyses. For further information see the ICCS technical report (Schulz et al., 2018b). The ICC of the scale is 0.078.

### Individual Level Background Variables

#### Societal Group

Members from the societally dominant ethnic group and members from societal minoritized groups were distinguished. These groups were based on the reported countries of birth of both parents. If at least one of the parents was born abroad, the student was classified as a member of a minoritized group (coded as 1). When both parents were born in the Netherlands, the student was classified as a member of the societally dominant ethnic group (coded as 0).

#### Gender

Gender was measured by self-report and coded as zero for boys and one for girls (50%).

#### Socio-Economic Background (SES)

SES was a composite variable based on the number of books at home, educational level parents, and occupation level parents (ISCO coded). It is provided by the ICCS consortium and calibrated to have a weighted mean of 0 and standard deviation of 1. The unweighted mean of the sample we use is 0.04 and the standard deviation is 1.

### Classroom Level Background Variables

#### Classroom Ethnic Diversity

Based on ethnic backgrounds of the students the Simpson Diversity Index (Simpson, 1949) was computed per classroom. Because for many ethnic groups the number of students was low, we created the following broader categories: native Dutch (N = 2184; 82%), Turkish (N = 58; 2%), Moroccan (N = 43; 2%), Surinamese, Antillean, Aruban (N = 74; 3%), Other European (N = 133; 5%), and Other non-European (N = 167; 6%). This index represents the chance that two students picked at random, have different ethnic backgrounds. This index ranged from 0.00 to 0.77 (*M* = 0.27, *SD* = 0.21). While diversity had quite a wide range, mean levels of diversity were rather low, suggesting that classroom diversity was low to moderate in most classrooms. However, the classroom diversity was only negligible skewed (0.63) and had little Kurtosis (−0.60).

### Analytic Strategy

The dataset was prepared with SPSS 27, and the analyses were conducted with Mplus 8.1 (Muthén & Muthén, 1998–2018). First, descriptive statistics are provided for the main study variables for societal dominant and minoritized ethnic group students. Second, in the main analyses, hypotheses were tested using multilevel analyses (Snijders & Bosker, 2012). Multilevel analyses are suitable because they take the nested structure of the data into account, and they can include individual level and classroom level variables simultaneously in the analyses. Two levels are distinguished: the student and the classroom level. As only one classroom per school participated in the study, the classroom level and school level are equivalent in this dataset.

Separate multilevel analyses were conducted for each dependent variable. Variables at the classroom level were centered at the grand mean. The dichotomous and continuous indicators of victimization were modeled as dependent variables in a two-part analyses using a Bayes estimator to enable their simultaneous consideration within the model. The two-part modeling approach improved the normality of the frequency of victimization scores over zero (no experiences of victimization). This procedure decomposes the original distribution of victimization scores into two parts. The first part of the model separates the zero victimization scores from the rest of the distribution of victimization scores (one or more victimization experiences). This binary score is analyzed as a logistic function with the log-odds of victimization regressed on the model factors. The second part is a traditional continuous multi-level model that is fit to the portion of victimization scores that is not zero. Fort this part, values of zero on victimization are treated as missing data. The two-part model can simultaneously estimate prevalence and frequency of victimization. The Bayes estimator allows the combined consideration of the dichotomous and continuous indicator when including cross level interactions. Other analyses use a maximum likelihood estimator. All models were built up stepwise, adding one group of variables in each subsequent model. The analyses employ Full-Information Maximum Likelihood (FIML) estimation of missing values. FIML uses all available data points to estimate parameters but excludes cases with missing values on covariates.

## Results

### Descriptive Statistics

Descriptive statistics are summarized in Table 1. These findings show that societally minoritized ethnic group members reported more experiences with peer victimization and experienced less positive peer relations at school than dominant ethnic group members. This indicates that minoritized group students had less positive social adjustment in school. Minoritized and dominant group students did not differ in their perception of the classroom climate for discussion.

**Table 1:** Means and standard deviations of peer victimization, positive peer relations, and open classroom climate for discussion for societal dominant group versus minoritized group members

	Societal dominant group members		Societal minoritized group members			
	*M*	*SD*	*M*	*SD*	*T*-value	*p*-value
Perceived peer victimization binary	0.61	0.49	0.70	0.46		
Perceived peer victimization sum	1.19	1.26	1.51	1.38	4.84	*p* < 0.001
Positive peer relations	3.17	0.47	3.06	0.53	−4.55	*p* < 0.001
Open classroom climate for discussion	47.36	8.60	47.45	9.03	0.20	*p* = 0.84

Societal dominant group members: *n* = 2168–2172; Societal minoritized group members: *n* = 470–474

*M* mean, *SD* Standard Deviation

Table 2 shows the student level correlations between the main study variables for societally minoritized group members and dominant group members. The socioeconomic status of dominant group members correlated slightly positively with positive peer relations and with their experience of an open classroom climate for discussion, and negatively with peer victimization. The socioeconomic status of minoritized students was not significantly related to peer victimization, positive peer relations, or their experience of an open classroom climate for discussion. Overall, the differences in the sizes of correlations between the groups were small. Tables 5 and 6 in the appendix give more detailed and level specific descriptive statistics for the study.

**Table 2:** Correlations of student level variables for the societal dominant group versus minoritized group members

	1.	2.	3.	4.	5.
1. Socioeconomic status	–	−0.011	−0.043*	0.111***	0.107***
2. Victimization binary	0.029	–	0.759***	−0.201***	0.023
3. Victimization sum	−0.018	0.704***	–	−0.312***	−0.002
4. Positive peer relations	0.075	−0.272***	−0.333***	–	0.077***
5. Open classroom climate for discussion	0.045	−0.049	−0.054	0.117*	–

Values of societal dominant group members above the diagonal (*n* = 2151–2169–2233) and values of societal minoritized group members under the diagonal (*n* = 461–474)

Unweighted correlations

*p > 0.05; **p > 0.01; ***p < 0.001

### Main Analyses

Before turning to the hypotheses, the multilevel findings regarding societal group differences (dominant versus minoritized) are discussed. These findings show that societal group differences in social adjustment also appear in multilevel regression analyses when gender, SES and the openness of the classroom climate are accounted for (Model 1 in Tables 3 and 4). When diversity was introduced in the next model (Model 2 in Tables 3 and 4) societal group membership (minoritized vs dominant group) was no longer significantly related to peer relations.

**Table 3:** Coefficients of multilevel analyses predicting experiences with victimization

	Model 1		Model 2		Model 3		Model 4		Model 5		Model 6	
	BIN	CON	BIN	CON	BIN	CON	BIN	CON	BIN	CON	BIN	CON
Student level												
Societal group (1 = dominant)	−0.100*	−0.063*	−0.101*	−0.042	−0.094*	−0.038	−0.100*	−0.027	−0.077*	−0.023	−0.095*	0.030
Gender (1 = girl)	−0.084*	−0.109*	−0.075*	−0.105*	−0.072*	−0.112*	−0.073*	−0.0116*	−0.068*	−0.109*	−0.076*	−0.117*
Socioeconomic status	−0.06	−0.066*	0.024	−0.057	0.070*	−0.007	−0.072	−0.005	0.072*	0.006	0.072*	0.005
Open classroom climate	0.020	−0.011	0.035	0.006	0.052	−0.003	0.049	−0.002	0.043	0.001	0.061*	0.020
R^2^	0.020	0.022	0.019	0.018	0.023	0.016	0.024	0.016	0.029	0.020	0.024	0.017
Classroom level												
Threshold / Intercept	−0.312	1.093	−1.220	7.167	−1.193	5.193	−1.225	4.937	^a^	5.559	−1.182	5.118
Socioeconomic status					−0.491*	−0.729*	−0.522*	−0.704*	−0.543*	−0.756*	−0.357*	−0.657*
Diversity			0.102	0.382*	0.057	0.107	0.062	0.238	0.020	0.020	0.040	0.251
Diversity squared							−0.026	0.087				
Open classroom climate for discussion											−0.292*	−0.267
Open classroom climate for discussion × Diversity											−0.032	0.105
Societal group × Diversity									−0.129	−0.019		
R^2^			0.015	0.146	0.259	0.616	0.298	0.595	0.305	0.646	0.292	0.687

Societal group: 0 = Minoritized, 1 = Dominant; Gender: 0 = boy; 1 = girls

*BIN* binary indicator of victimization, *CON* continuous indicator of victimization

*p < 0.05

^a^not provided; standardized parameters; Bayes estimators

**Table 4:** Coefficients of multilevel analyses predicting positive peer relations

	Model 1	Model 2	Model 3	Model 4	Model 5^a^	Model 6
Student level						
Societal group (1 = dominant)	0.080***	0.040	0.040	0.039	^b^	0.040
Gender (1 = girl)	−0.023	−0.024	−0.024	−0.025	−0.023	−0.024
Socioeconomic Status	0.097***	0.058**	0.013	0.013	0.007	0.013
Open classroom climate for discussion	0.078***	0.071***	0.064**	0.064**	0.003*	0.062**
R^2^	0.025	0.011	0.006	0.006	^b^	0.006
Classroom level						
Intercept	0.000	−0.042	−0.013	0.032	−0.009	−0.014
Socioeconomic status			0.586***	0.573***	0.158***	0.577***
Ethnic diversity		−0.378***	−0.322***	−0.293*	−0.226**	−0.317**
Ethnic diversity squared				−0.055		
Open classroom climate for discussion						0.016
Open classroom climate for discussion * Ethnic diversity						0.042
Societal group * Ethnic diversity					−0.202	
R^2^ Level 2		0.143	0.478	0.481	^b^	0.482

Standardized estimators, ML estimator

Societal group: 0 = Minoritized, 1 = Dominant; Gender: 0 = boy; 1 = girl

*p > 0.05; **p > 0.01; ***p < 0.001

^a^Unstandardized estimators

^b^Not available with the two level random option

It was hypothesized that classroom diversity would be related to better social adjustment among minoritized group students (Hypothesis 1a) and to worse social adjustment among dominant group students (Hypothesis 1b). To test Hypotheses 1a and 1b, both the direct effect of classroom ethnic diversity and the interaction term of classroom ethnic diversity with societal group (minoritized vs dominant) are relevant. Classroom diversity was related to more peer victimization and less positive peer relations at school (see Model 2 in Tables 3 and 4). When introducing classroom SES (latently aggregated in Model 3), the negative effect of classroom diversity on positive peer relations remained stable while classroom diversity no longer explained peer victimization. Model 5 introduced the cross-level interaction of societal group (minoritized vs dominant) with classroom diversity, which was not significant. This means that the relation between classroom diversity and the two aspects of social adjustment did not differ between minoritized and dominant group students. Thus, in line with Hypothesis 1b, classroom diversity was related to less positive social adjustment among societally dominant ethnic group members. Contrary to Hypothesis 1a, this was also the case for societally minoritized group members. Furthermore, classroom diversity and the measures of social adjustment were not found to have a curvilinear relationship (Model 4 in Tables 3 and 4).

Next, it was hypothesized that classroom ethnic diversity would be more positively related to social adjustment in classrooms with an open classroom climate for discussion (Hypotheses 2). To examine Hypotheses 2, Model 6 introduced the open classroom climate for discussion on the classroom level as well as the interaction of the classroom climate for discussion and classroom diversity. The non-significant interaction effect shows, contrary to Hypothesis 2, that classroom diversity does not moderate the relation between classroom diversity and students’ social adjustment.

### Robustness Checks

To investigate the robustness of the analyses, further analyses were conducted (see Appendix B). First, the relevance of educational track was assessed. Educational track of classrooms was obtained from schools during data collection. This was dummy coded into 0, *lower track (pre-vocational)*, and 1, *middle or higher track (general)*. 51% of the students visited the general track. Educational track was added to the model along with classroom diversity and classroom SES. Educational track was no predictor for positive peer relations at the classroom level but in the general tracks students experienced less peer victimization (see Model 1 in Tables 7 and 8). Including educational track did not change the relevance of classroom diversity.

Secondly, the approach by Smith et al. (2016) was replicated, who did not use the Simpson Diversity Index (SDI) but used the percentage of societally dominant group students along with an SDI that excluded dominant students. This resulting index is termed immigrant diversity and indicates the diversity among different minoritized groups (see Model 3 in Tables 7 and 8 in the appendix). While the proportion of dominant group students within a classroom was related to more positive peer relations, immigrant diversity was not related to positive peer relations. The association between the proportion of dominant group students and peer relations is unsurprising because the proportion of dominant students nearly perfectly correlates with (total) classroom diversity (*r* = 0.935; *p* > 0.001). These analyses suggest that when controlling for the proportion of dominant students the amount of diversity among minoritized groups has no additional association with social adjustment.

## Discussion

An increasingly diverse school going population asks for insight into how ethnic diversity in the school context affects the lives of students. How diversity affects students’ social adjustment at school is particularly relevant because these experiences affect both students’ learning and achievement. Whereas several studies in the United States showed that school and classroom ethnic diversity were related to better social adjustment of students (e.g., Juvonen et al., 2018; Graham, 2018), research on this association in European classrooms is scarce and limited in scope. Hence, the current study examined how classroom diversity in Dutch classrooms was related to social adjustment of students from societally dominant versus minoritized ethnic groups, and the potential moderating role of an open classroom climate for discussion in this association.

In contrast with earlier findings in the U.S., and in line with previous studies on school bullying and victimization in the Netherlands, the current results from Dutch classrooms showed that classroom ethnic diversity was related to less positive social adjustment (more peer victimization, less positive peer relations). The negative association was especially pronounced and stable for peer relations. Whereas the results show that, overall, minoritized group students experienced worse peer relations at school than dominant group students, the relation between classroom ethnic diversity and social adjustment did not differ between both groups. This means that classroom diversity was related to less positive social adjustment irrespective of societal group membership. Also, classroom diversity was related to less positive peer relations, beyond the effects of educational track and SES while the association between diversity and victimization seemed to be fully accounted by SES. The additional analyses suggest that the negative association between classroom diversity and social adjustment is largely driven by a lower proportion of societally dominant students (i.e., a higher proportion of students from minoritized groups) in ethnically more diverse classrooms.

One explanation for why the results of the current study were not in line with the more optimistic studies from the US, are the relatively low levels of ethnic diversity within Dutch classrooms. Regarding the balance of power hypothesis, it should be noted that if we look at the range of ±1 SD of the mean of Simpson’s diversity index, then 68,27 % of students in the examined sample attended classrooms where diversity did not exceed 0.50. This means that the balance of power principle may still hold but we could not test it because balance of power was not achieved in these mostly low-to-medium diverse classrooms. What we can conclude, however, is that when examining the levels of diversity at hand here (i.e., generally low to medium levels), higher ethnic diversity is related to less positive social adjustment. Moody (2001) has shown that moderate diversity actually increases ethnic homophily. It can be argued that moderately diverse school environments make ethnicity particularly salient by highlighting group distinctions and thus leading to more negative peer relations (Bellmore et al., 2012) and a negative race/ethnic climate (Benner & Graham, 2013). Some studies suggest that students from the societally dominant group may feel threatened by higher numbers of students from minoritized groups in the classroom, and hence resort to stronger in-group affiliation in moderately diverse classrooms as long as they still represent the numerically dominant group (Grütter et al., 2021; Smith et al., 2016). This was also documented by a recent study in the Netherlands (with similar levels of classroom diversity as in the current study) that showed that in more ethnically diverse classrooms, students particularly turn to their ingroup peers (Munniksma et al., 2017b).

It is noteworthy that the relationship between classroom diversity and social adjustment was partly explained by mean levels of classroom SES. Diversity was still associated with less positive peer relations, but not with more victimization, when the classroom SES was accounted for. There are two potential explanations for this. One is that classroom diversity in our sample was highly negatively correlated with the percentage of dominant group students and that schools with a higher proportion of minoritized students are often concentrated in less affluent neighborhoods in the Netherlands. As shown in Table 6 of the Appendix classroom level socio-economic status is also positively correlated with the proportion of dominant group students (*r* = 0.23). This means that some of the negative effects of classroom ethnic diversity observed in this study may be due to concentrations of less affluent students. Low SES is a well-known risk factor for social adjustment (Jansen et al., 2012). Alternatively, given that SES could not account for the relationship between diversity and positive peer relations, the non-significant relationship between diversity and peer victimization frequency could also be due to a lack of power. This is because this variable only refers to those adolescents who experienced any victimization at all and the descriptive statistics presented in Table 1 suggest that between 30% (minoritized group) to 39% (dominant group) of adolescents were never victimized.

Another point to consider is the examined age group, which were middle adolescents (aged 14) while other research on the balance of power hypothesis has focused on early adolescents (e.g., Juvonen et al., 2018). In part related but more important than the age group may be to what extent students have already become accustomed to ethnic diversity in their classrooms. It is likely that most dramatic effects of diversity can be observed when students transition from primary to secondary school, especially when both contexts differ in diversity. In this study, students were in their second year of secondary school and there is research to suggest that over time diversity effects may dissipate as students get used to their surroundings (e.g., Jugert et al., 2011).

While our findings did not show positive outcomes of diversity we did not examine outcomes related to intergroup relations. Even though classroom diversity may create challenges for group dynamics, it also creates the opportunity to learn to deal with differences (Sincer et al., 2021). Also, classroom diversity has been shown to be related to improved intergroup relations and attitudes (e.g., Bohman & Miklikowska, 2020). Research also indicates that positive social dynamics (e.g., having positive intergroup contacts) are beneficial for a positive relation between classroom diversity and outgroup attitudes (Bekhuis et al., 2013; Stark et al., 2015; Janssen et al., 2016). These social dynamics can be worse in moderately diverse classrooms, as our study shows. This points to the importance of fostering positive intergroup dynamics in moderately diverse classrooms.

The current study points out that an open classroom climate for discussion can improve social adjustment of students, but it did not affect the link between classroom diversity and social adjustment. The literature suggests different alternative ways in how to manage ethnic diversity within the classroom context (Grutter et al., 2021; Schwarzenthal et al., 2020). The findings of the current study indicate that the importance of investing in intergroup dynamics is high. While open classroom climate for discussion has been shown to improve students’ civic knowledge (Torney-Purta et al., 2001) and tolerance toward outgroup members (Janmaat, 2012) it is important to note that open classroom climate for discussion does not necessarily involve an explicit discussion about issues of ethnic diversity with students. More recent concepts like school racial climate (Byrd, 2015) and cultural diversity climate (Schachner et al., 2016) suggest that it may be necessary for teachers to explicitly foster contact and cooperation between students from different ethnic groups but also to value cultural pluralism and raise students’ critical consciousness about societal inequalities. Unfortunately, existing studies have shown that teachers are ill-prepared for an active management of ethnic diversity and often prefer a color-blind approach that stresses similarities rather than acknowledging and valuing differences to dealing with diversity (e.g., Civitillo et al., 2017).

Several limitations should be noted. First, the cross-sectional nature of this study does not allow conclusions about causality. Therefore, longitudinal studies are warranted even though reverse causality affecting a structural factor like school diversity is highly unlikely. Second, the range of diversity within the classrooms was limited to the diversity levels in Dutch classrooms which ranges from low to medium. Thus, there is a limited number of classrooms in which there is high diversity and theoretically, a balance of power. To examine such balance of power in the Netherlands, and to extent the generalizability to schools in more (urban) multicultural areas, future studies should oversample classrooms with higher ethnic diversity (see e.g., Agirdag et al., 2011; Smith et al., 2016). However, it should be noted that the sampled schools are a representative sample of schools in the Netherlands. The limited variance of diversity in the sample therefore should reflect the limited variance in Dutch schools. Third, whereas we focused on whether sharing perspectives within an open discussion climate would promote a positive link between classroom diversity and social outcomes, future studies should examine other ways by which teachers can promote positive outcomes of diversity. Promising directions in this regard are studies focusing on student-teacher relationships (e.g., Civitillo et al., 2021; Grütter et al., 2021) or on active diversity management by teachers (e.g., Schwarzenthal et al., 2020).

Nonetheless, to findings of the study are important for educational practice. Because diversity is related to worse social adjustment, and social adjustment is important for the academic and social development of adolescents, specific attention should be paid to the social dynamics within ethnically diverse classrooms. Given the importance of preparing students to become part of a pluralistic society, it is regrettable that teacher training in the Netherlands pays little attention to diversity (Severiens et al., 2014), and that accordingly a substantial number of beginning teachers feel inadequately prepared to handle the challenges of urban multicultural classrooms (Gaikhorst et al., 2017).

## Conclusion

Previous research from the US found classroom ethnic diversity to be related to more positive social adjustment of students. European studies on this topic are limited in scope and inconclusive. Based on nationally representative data, the current study adds to previous research on the relation between classroom diversity and students’ social adjustment. In contrast to studies in the US, classroom diversity was related to worse social adjustment in low to moderately diverse classrooms in the Netherlands. This was found for students from the societally dominant group and from minoritized groups. This finding was most robust for positive peer relations, even after controlling for SES and track. The association between classroom diversity and peer victimization was explained by classroom SES, which was strongly related to the social adjustment of students. The current study underscores the need for attention to social dynamics in schools with a low to moderately diverse student population, in both teacher training and practice. Simply placing students with different backgrounds together in a classroom does not inevitably lead to better social outcomes. An open classroom climate for discussion may be used to foster positive social adjustment in general, but an open classroom climate for discussion is not enough to ensure a nurturing social context for diverse classrooms.

## Appendix A - Additional Descriptive Statistics

Tables 5 and 6

**Table 5:** Multilevel descriptive statistics after grand mean centering

Variable	Mean within	Variance within	Mean between	Variance between	Intraclass correlation
Gender (1 = girl)	0.000	0.02	0.00	0.01	0.020
Socioeconomic status	0.000	0.71	−0.05	0.30	0.296
Societal group (1 = dominant)	0.000	0.12	0.00	0.03	0.183
Positive Peer Relations	0.000	0.21	−0.01	0.02	0.094
Victimization sum	0.000	1.59	0.01	0.09	0.059
Victimization binary	0.000	0.23	0.00	0.01	0.037
Open classroom climate for discussion	0.000	69.69	−0.03	5.90	0.077
Total Diversity			0.00	0.04	

*N* = 2703 students in 119 schools

**Table 6:** Correlations of study variables on the between level

	(1)	(2)	(3)	(4)	(5)	(6)
(1) Socioeconomic status						
(2) Positive Peer Relations	0.610***					
(3) Victimization sum scale	−0.611***	−0.951**				
(4) Open classroom climate for disc.	0.444***	0.281**	−0.442***			
(5) Total Diversity	−0.088	−0.367***	0.364**	−0.015		
(6) Proportion dominant students	0.226*	0.443***	−0.394***	0.093	−0.935***	
(7) Immigrant diversity	0.027	−0.229*	0.237*	−0.042	0.758***	−0.636***

*N* = 2703 students in 119 schools

**p* < 0.05; ***p* < 0.01; ****p* < 0.001

## Appendix B – Robustness Checks

Tables 7 and 8

**Table 7:** Supplementary analyses of victimization

	Model 1		Model 2		Model 3		Model 4	
	BIN	CON	BIN	CON	BIN	CON	BIN	CON
Student level								
Societal group (1 = Dominant)	−0.103*	−0.029	−0.099*	−0.026	−0.102*	−0.033	−0.102*	−0.038
Gender (1 = girl)	−0.071*	−0.111*	−0.077*	−0.113*	−0.082*	−0.107*	−0.077*	−0.108*
Socioeconomic status	0.071*	0.007	0.053*	−0.010	0.058*	−0.010	0.052	−0.011
Open classroom climate for discussion	0.052	0.007	0.048	001	0.051	−0.001	0.049	−0.002
R^2^	0.025	0.015	0.023	0.015	0.025	0.015	0.023	0.016
Classroom level								
Threshold / Intercept	−1.276	5.959	−1.042	4.681	−1.071	4.891	−1.249	4.532
Socioeconomic status	−0.273	−0.461*	−0.523*	−0.697*	−0.513*	−0.636	−0.531*	−0.680*
Diversity	0.083	0.328	0.542	0.677				
Track (1 = general)	−0.384*	−0.435*						
Proportion dominant students			0.468	0.405	0.057	−0.197	−0.029	−0.336
Immigrant Diversity			−0.026	0.084	0.102	0.187	0.003	0.366
Proportion dominant students squared							−0.027	−0.382*
Immigrant diversity squared							−0.127	0.307
R^2^	0.283	0.600	0.348	0.695	0.311	0.575	0.357	0.676

*BIN* binary indicator of victimization, *CON* continuous indicator of victimization

**p* < 0.05; Standardized parameters; Bayes estimators

**Table 8:** Supplementary analyses of positive peer relations

	Model 1	Model 2	Model 3	Model 4
Student level				
Societal group (1 = Dominant)	0.041	0.039	0.039	0.040
Gender (1 = girl)	−0.025	−0.028	−0.028	−0.028
Socioeconomic Status	0.013	0.025	0.025	0.025
Open classroom climate for discussion	0.063**	0.058**	0.058**	0.058**
R^2^	0.006	0.006	0.006	0.006
Classroom level				
Intercept	−0.025	−00.028	−0.030	−0.162
Socioeconomic status	0.480*	0.550***	0.527***	0.530**
Diversity	−0.327*	−0.322		
Track (1 = general)	0.143			
Proportion of societal dominant students		0.042	0.309*	0.459*
Immigrant diversity		−0.047	−0.119	−0.035
Proportion of societal dominant students squared				0.140
Immigrant diversity squared				0.046
R^2^	0.485	0.536	0.527	0.543

Unstandardized estimators; *N* = 2703 students in 119 schools

ML Estimators

**p* < 0.05; ***p* < 0.01; ****p* < 0.001
